# Consensus Paper: Situated and Embodied Language Acquisition

**DOI:** 10.5334/joc.308

**Published:** 2023-10-10

**Authors:** Lorraine D. Reggin, Ligia E. Gómez Franco, Oleksandr V. Horchak, David Labrecque, Nadia Lana, Laura Rio, Gabriella Vigliocco

**Affiliations:** 1 University of Calgary, Calgary, AB, Canada; 2 Ball State University, Muncie, IN, USA; 3 Iscte-Instituto Universitário de Lisboa, CIS_iscte, Lisbon, Portugal; 4 Universitédu Québec àMontréal, Montréal, QC, Canada; 5 McMaster University, Hamilton, ON, Canada; 6 Universitàdi Bologna, Bologna, Italy; 7 University College London, London, UK

**Keywords:** language acquisition, language learning, embodiment, situated context

## Abstract

Theories of embodied cognition postulate that perceptual, sensorimotor, and affective properties of concepts support language learning and processing. In this paper, we argue that language acquisition, as well as processing, is situated in addition to being embodied. In particular, first, it is the situated nature of initial language development that affords for the developing system to become embodied. Second, the situated nature of language use changes across development and adulthood. We provide evidence from empirical studies for embodied effects of perception, action, and valence as they apply to both embodied cognition and situated cognition across developmental stages. Although the evidence is limited, we urge researchers to consider differentiating embodied cognition within situated context, in order to better understand how these separate mechanisms interact for learning to occur. This delineation also provides further clarity to the study of classroom-based applications and the role of embodied and situated cognition in the study of developmental disorders. We argue that theories of language acquisition need to address for the complex situated context of real-world learning by completing a “circular notion”: observing experimental paradigms in real-world settings and taking these observations to later refine lab-based experiments.

In this paper, we address the link between situated cognition and embodied cognition during the course of language acquisition. From our perspective, embodied cognition describes a theoretical position according to which our internal cognitive mechanisms are grounded into our sensorimotor and affective systems. In this view, for example, understanding the word “cooking” implies simulation of motor and sensory aspects of the real-world experience of cooking (and/or watching a Jamie Oliver cooking show). For these aspects to be simulated, they need to have been experienced in our actual experiences (in the “here and now”) especially during acquisition so as to become part of our semantic representations. We refer to the “in-the-moment” processes that occur within the environmental context as situated cognition. The situated properties can include cultural norms, the physical environment, the social contexts of communication, as well as the discourse context.

Theories of embodied cognition suggest that modality-specific systems for perception, action and emotion are involved in language processing (Barsalou, 1999; Vigliocco et al., 2009; Wilson, 2002; Zwaan, 2004). While empirical evidence in support of this view has primarily focused on adult cognition (see Horchak et al., 2014, for a review), a growing body of research suggests that children also rely on sensorimotor simulations to acquire vocabulary and develop conceptual knowledge (see Wellsby & Pexman, 2014a, for a review).

The ability for children to engage in sensorimotor simulations must arise from an interaction between the developing sensorimotor and affective systems and the external world, including the physical, social, and broader cultural environment with which the child interacts. The physical environment includes objects that can be perceived and manipulated as well as trigger specific emotions. The social and cultural environment includes caregivers, teachers, and peers who help develop mental representations from the sensorimotor and affective experiences of the child. Language acquisition is always embedded in a face-to-face context: there are objects acted upon and talked about, and caregivers providing additional cues, such as gestures, that can bolster language development (Vigliocco et al., 2019) in a manner that can be culturally specific.

The process by which the sensorimotor and affective properties of object, actions, etc become part of the conceptual representations for the corresponding objects, actions etc. and therefore these properties become the basis for simulation must come about in the *situated* context in which the learning occurs. Let us walk through an example of how the situated context can support the development of simulations. Imagine a child going to the park with their caregivers and for the first time seeing, riding a merry-go-around and having a lot of fun. The caregiver also tells the child the name of the object (merry-go-around) while they are riding and laughing. In this situated context, the child therefore begins to map the label “merry-go-around” with the experience of how the object looks, how you use it and the associated emotions. Imagine then the caregiver a day later at home saying to the child “it was fun to ride the merry-go-around!” while smiling and making an iconic gesture moving their index finger in circles. The presence of the gesture and the positive emotion expressed can help the child build stronger associations between the label and the object by virtue of evoking the physical experiences they had before. Here, the situated context does not involve the presence of the physical object, but involves the communicative/social context of the caregiver-child interaction in which elements of the sensori-motor and affective experience are enacted. Both the physical as well as the social/communicative context, therefore, can ground the development of the sensori-motor and affective associations underscoring simulations (see Murgiano et al., 2021, for a description and discussion of situated language and its importance to language development).

The interdependence between embodied and situated aspects is not only used/useful in development. In adulthood, we also see important interactions. For example, comprehending language about visually accessible events such as watching a person describing how to build an IKEA shelf while actually building it, may requires different (arguably less) simulations that comprehending language about the same event without seeing a person building it (Zwaan, 2014). Even when the description is not accompanied by the actions, simulations may differ in the context in which we see the speaker gesturing as if they were building the shelf or we only hear the description over a phone line (or read it). Thus, the situated context, being the actual physical presence of the objects and actions talked about, or the enactment by a speaker in their communication of the objects and actions talked about can impact comprehension processes via the type (and arguably amount) of simulation required.

Despite this interdependence, most of the previous literature has considered embodied and situated cognition as separate and focused only on one or the other. For example, in the adult literature, studies of embodiment focus on decontextualized language processing (e.g., Meteyard et al., 2007; Pulvermüller et al., 2001), while studies of situated language processing focus on processing in a (predominantly) visual context (e.g., Huettig et al., 2011; Knoeferle & Guerra, 2016; Tanenhaus et al., 2000).

In this position paper, we provide key examples from the literature (both adults as well as children), to highlight the limitations of considering embodied cognition and situated cognition as separate and independent processes and the promise of using an approach in which embodiment is brought back to the situational context. First, although clearly research can focus more on embodiment *or* situated language, these two aspects cannot be considered as independent and separate: language acquisition and processing is *always* situated, and the degree of embodiment may dynamically change depending upon *how* language is situated. Second, it is the situated nature of language (and conceptual) acquisition that enables embodiment.

## Embodied language is situated in adulthood

In healthy adults, the brain network recruited by speech processing includes a regular contribution of the motor areas (Skipper et al., 2017). The phenomenon takes place irrespective of the modality of the speech stimuli, testifying to a strict action/perception association that fits well with the contemporary neuroscientific frameworks positing shared representations for actions and the perception of these actions (Hommel et al., 2001; Rizzolatti & Fabbri-Destro, 2009). It follows that contemporary investigations of phonological development cannot neglect motor processes. The role of the motor system in language comprehension may be less central than advocated by Glenberg (2015), based on the failure to replicate action sentence compatibility effects (ACE) in a large pre-registered, multi-lab study (Morey et al., 2022). However, many studies using body-object interaction norms (BOI; the degree to which a concept can be interacted with physically) support the involvement of the motor system in comprehension, along the lines suggested by weak embodiment proposals (e.g., Meteyard et al., 2012; Vigliocco et al., 2009). A BOI effect (Siakaluk et al., 2008), demonstrated that adults are faster and more accurate in semantic categorization and lexical decision tasks when responding to words with referents that the human body can more easily interact with the word’s referent (Pexman et al., 2008; Siakaluk et al., 2008; Wellsby et al., 2011; Yap et al., 2011). In addition, fMRI studies (Dreyer & Pulvermüller, 2018; Hauk et al., 2004; Tettamanti et al., 2005) have shown activation of motor areas in response to action verbs (e.g., kick) corresponding to the associated physical action (e.g., leg).

Embodiment effects have also been demonstrated for visual perception. For example, Meteyard and colleagues (2007; 2008) provided evidence that comprehending words referring to upward and downward motion recruit the visual motion perception system. Meteyard et al. (2007) and Meteyard et al. (2008) discovered that words referring to upward and downward motion activate the visual motion perception system. They used a perceptual task to demonstrate that incongruent verbs impaired perceptual sensitivity and found that lexical decision times were slower for motion words when incongruent with threshold-level motion displays of dots.

Although much research efforts have been devoted to action and visual systems, there is also empirical evidence for the simulation of such other sensory modalities as touch, taste, and olfaction during language comprehension (see Speed & Majid, 2020, for a comprehensive review). With regards to touch, Brunyé et al. (2012) instructed participants to rate fabric samples after reading sentences that described tactile (e.g., “try a pair of thick corduroy pants”) or non-tactile (e.g., “own many pairs of pants”) experiences. A major result was that the ratings were higher whenever there was a compatibility between the texture (rough vs. smooth) of the fabric implied by the sentence and the texture of the actual fabric sample. This result suggests that participants simulated the textile experience during sentence reading. With regards to taste, Pecher et al. (2003) asked participants to verify concept-property pairs (e.g., lemon-sour) and found that participants verified the second property faster when it came from the same modality as the first property (e.g., cranberries-tart) than when it came from a completely different sensory modality (e.g., blender-loud). Thus, a processing cost from switching from one modality (e.g., taste) to another (e.g., audition) shows that language comprehenders recruited gustatory representations for taste-related concepts. With regards to olfaction, González et al. (2006) had participants read odour-related words (e.g., jasmine, garlic, etc.) and found that reading words that bear strong olfactory associations activates olfactory regions of the brain. This points to the conclusion that olfactory representations may also get activated during language comprehension.

The studies above focused on processing single isolated words (or short decontextualized sentences), therefore ignoring the situational context in which their (more or less) embodied representations are used. At a very general level, this is problematic because such approaches may lack ecological validity (see Vigliocco et al., 2023, for a discussion). The importance of including some aspects of context has been highlighted by Zwaan (2014) who argues that the magnitude of these effects may vary depending on the environmental context. He proposes that the degree of simulations required depends on the specific context and purpose of comprehension. He argues for levels of embeddedness that are determined by the communicative and referential situated context. When comprehending discourse, adults simulate a situation complete with agents, objects, and location. The degree of embeddedness in the physical environment determines how much the person will need to rely on their memory to activate the representation (see Horchak & Garrido, 2022; Wu & Barsalou, 2009; Yaxley & Zwaan, 2007, for empirical evidence on how representations of objects are integrated with representations of described environmental context). The degree of embeddedness will also depend on the person’s goals during comprehension.

Snefjella and Kuperman (2016) found that words tend to absorb the valence of the surrounding linguistic context, and that this acquired valence impacts processing. This finding was experimentally tested in Snefjella et al. (2020) using a novel word learning paradigm, where novel words were embedded in linguistic contexts with varied valence, and after reading, participants were tested on the quality of their orthographic and semantic learning of the novel words. Valence of the linguistic context contributed to learning, with novel words occurring in linguistic contexts with a positive valence showing advantages in semantic learning compared to negative or neutral contexts. Furthermore, affective and sensorimotor dimensions of words have both been found to play independent roles in adult word learning (Espey et al., 2023; Lana & Kuperman, in press), suggesting that the interaction of these dimensions might diminish with age (see discussion on children’s abstract language acquisition below). Linguistic context contributes to the learning environment, which is hypothesised to generate bodily and emotional experience that is encoded when learning and simulated when processing the word.

In addition, language is rarely constrained to a single mode of communication but rather is situated within a multimodal interaction (Murgiano et al., 2021). The speaker provides additional information that may aid the comprehension of the communicative partner by assisting with semantic retrieval. Gestures, body and face movements, eye gaze, and more, can support the processing. For example, speakers can point or look to objects in the environment while talking about them, they can represent properties of the referents using iconic gestures (e.g., moving a fist up and down while talking about hammering), and they can use iconic prosody (e.g., saying “it was a loooong trip” when talking about their return trip from holiday). For example, Zdrazilova et al. (2018) asked participants to communicate the meaning of a word to a partner without using the word itself to analyse the speech and gestures used by the participants to communicate the meaning to their partner. The results showed that participants drew on several aspects of the meaning to convey the concept to their partner. Gesture analysis indicated that participants used metaphorical gestures (hand movements that represent abstract ideas in an imagistic manner, e.g., moving the hand up while talking about inflation) and beat gestures (i.e., repetitive hand movements time-locked to the rhythm of the speech) to communicate abstract word meanings but iconic gestures (movements that imagistically depict aspects of a concrete referent; e.g., holding hands in the shape of a round ball) to convey concrete word meanings. Gestures are an integral component of speech during naturalistic face-to-face communication, they are produced and comprehended automatically, and integrated with the speech. Demonstration of such integration comes from work showing that listeners cannot help but be affected by gestures even when these are irrelevant for the task. For example, in a study by Kelly et al. (2010), it was found that participants were less accurate and had slower reaction times in matching pictures of actions to words when incongruent gestures (e.g., word “cut”, gesture “pulling”) were presented with the words. The degree of incongruency also affected processing, the participants had more errors for strong incongruities (e.g., speech: “chop”; gesture: “twist”), indicating that adults are highly sensitive to the gestural context during language processing (see also Perniss et al., 2020). It is not only gestures, but also other manual actions, that support language learning. For example, a study by Macedonia et al. (2020) found that for L2 speakers, grasping an object aided in the learning of pseudowords referring to the objects, pointing to a role of sensorimotor information in language learning.

## Embodiment effects emerge late in development

Within the last decade, some evidence for embodied effects of perception and motor systems in children’s language acquisition have emerged, though the picture is not always clear. Hupp et al. (2020) demonstrated that as early as during the preschool years, children visually simulate shape information implied by a linguistic context, thus providing further credence to the claim that embodied representations are routinely activated during language processing. In a different example, Vogt et al. (2019) provided evidence that children (aged 4 to 8 years old) mentally simulate target objects in the lower and upper space congruent to the object’s spatial location in the physical world (e.g., lower space for shoe vs. upper space for sun). In older children, there is also evidence for perceptual simulations. Engelen et al. (2011) showed that 7 to 13-year-old children construct a perceptual simulation of an object’s shape (e.g., an intact shape vs. a deformed shape) when they listen to or read sentences like “John saw an egg in a skillet/John saw an egg in a box”. Interestingly, de Koning, Wassenburg, et al. (2017) completed a perceptual simulation task during language comprehension and tested 3 groups: an adult group, a group of 9–10 year old children and a group of 11–12 year old children. Participants read a sentence about an object (e.g., “She looked at the bone of a rabbit/dinosaur”) with either an implied “small” or “large” size. Then they were shown a picture (small or large) and asked to respond “yes” or “no” whether the object was mentioned in the sentence. The participants responded faster when the size of the object was congruent to the implied size from the sentence. They concluded that children activate visual information on object size. Interestingly, although the adults responded faster to the pictures than the children, the pattern of the effects was the same. Furthermore, research suggests that children may represent not only static but also dynamic perceptual events. For example, Hauf et al. (2020) and Seger et al. (2020) demonstrated that children’s responses to pictured objects on a computer screen were faster when the objects moved in the directions that matched the linguistic context. Consistently, there is evidence, similar to the adult literature, of embodied effects of perception on language processing in children.

Wellsby and Pexman (2014b) investigated the BOI effect in younger (6–7 years) and older (8–9 years) children in a word naming task. They found that only older children were faster to name high BOI words than low BOI words indicating richer semantic representations for words with higher BOI. However, using a different task (auditory word repetition), Inkster et al. (2016) found a BOI effect for 6- to -7-year-old children. Furthermore, Inkster et al. (2016) also observed an imageability effect in auditory word repetition, consistent with the evidence that children engage perceptual simulations of object shape and size information that was described at the beginning of this section. More recently, Muraki et al. (2022) collected child BOI ratings (ratings of children’s sensorimotor experience with objects) and found that these ratings were more strongly related to valence and sensory experience than were adult BOI ratings.

Importantly, Wellsby and Pexman (2019) conducted two experiments testing the degree to which sensorimotor experience modulated 5-year old children’s language acquisition. Contrary to the predictions of an embodied account of language acquisition, there was no effect of the degree of sensorimotor experience between learning conditions that varied in amount of sensorimotor information (see Pexman, 2019, for a comprehensive review of the evidence of embodied effects in conceptual development). Given these findings, one may argue that there is no need for sensorimotor simulation for word and concept acquisition. However, what these findings may also suggest is that embodied aspects of semantic/conceptual representations require a lengthy period to develop in which direct, situated, experience is critical.

Valence has also been shown to support children’s acquisition of word meanings, and in particular meanings of words low in concreteness (i.e., abstract words). Several studies have demonstrated the role of valence in grounding the acquisition of abstract concepts (see Lund et al., 2019; Ponari et al., 2018; Wellman et al., 1995), supported also in part by linguistic information (Andrews et al., 2009; Gleitman et al., 2005), the situated context (Barsalou & Wiemer-Hastings, 2005), and interoceptive strength (Lynott et al., 2020; Reggin et al., 2021).

Ponari et al. (2018) and Lund et al. (2019) tested the impact of word valence on children’s lexical processing in an auditory lexical decision task. Ponari et al. (2018) tested 6–7-year-old, 8–9-year-old, and 10–11-year-old children using words with positive, neutral, and negative valence and measured accuracy, ensuring that the children at different ages knew those words (based on AoA). Lund et al. (2019) tested a younger group (5-, 6-, and 7-year old children) with a larger set of words specifically chosen to be familiar to children in the age range (AoA *M* = 5.35). Ponari et al. (2018) found a significant effect of valence on accuracy rates but only in the 8–9 year-old group and only for abstract words. Lund et al. (2019) extended the findings by testing a younger age group with more words and earlier-acquired words. They did not find an effect of valence in the 5-year-olds in either reaction time or response accuracy. They did, however, find that positive valence was facilitatory for abstract words, but not for concrete words in 6-year-olds as measured by reaction times, but the effect on accuracy data did not reach significance. As well, 7-year-old children responded more quickly to positive than neutral words, irrespective of concreteness.

All these studies support the idea that the *internal* sensorimotor and affective mechanisms that are evident in adult language processing are also at play in development. Grounding can provide the child with key aspects of meaning on which to anchor their representation of the words they are learning. One problem with these studies, however and again, is their lack of ecological validity. They do not assess the learning and processing of words/concepts in conditions similar to those in the real-world: they assess knowledge of single words in artificial tasks, and therefore strip away the learning and processing from the situated context in which learning and processing typically occurs. Language, especially language used with children, tend to refer to the “here-and-now”, namely to the physical setting in which it is used; and most of all, language is learnt and used in face-to-face interaction.

Crucially, in development, these effects are not found (or have not been studied) in younger children. This may certainly be in part due to difficulty in creating tasks and materials that can successfully tap into embodied semantic/conceptual representations in infancy or early childhood. Yet, we argue here, it may also offer some hints to the developmental trajectory of embodied representations from multiple situations. Acquiring adult-like embodied meanings for words requires multiple situational experiences that are amassed from birth through early childhood.

## Language and conceptual acquisition are necessarily situated before becoming embodied

Early years offer plenty of situational experiences that can be internalised. There is a large body of evidence that both quantity and quality of linguistic input predict children’s early language acquisition (e.g., Adamson et al., 2015; Anderson et al., 2021; Pereira et al., 2014). Quantity of linguistic input in early childhood predicts early language outcomes as well as later cognitive and language outcomes (Gilkerson et al., 2018; Golinkoff et al., 2019; Hart & Risley, 1992; Hjetland et al., 2019; Suggate et al., 2018). For example, Gilkerson et al. (2018) found that the number of adult words heard by the child (both overheard and child-directed speech) between the ages of 18–24 months predicted the child’s verbal comprehension index at age 9–13. Even more strikingly, the conversational turn count (a measure of adult-child initiation and responses that occur within 5 seconds) predicts age 9–13 child IQ, verbal comprehension index, and vocabulary scores even when controlling for maternal attained education as a marker for socio-economic status (SES). With respect to quality of adult input, Hsu et al. (2017) found that verb diversity in parent input to toddlers predicted child verb production six months later (see also Cartmill et al., 2013).

Not only the linguistic context matters. The situated physical context describes to what extent the learning and processing of language is supported by information in the “here and now”. Most research on early word learning assumes that the learning occurs only when the actual referent, an object or an action, is present or unfolding while the corresponding word is produced by a caregiver (but see Motamedi et al., 2022). When the physical context is cluttered (i.e., the child may be experiencing several objects unknown to them when a specific label is used), mechanisms such as cross-situational learning (Smith et al., 2007) may support the child to learn new words present in the scene. Cross-situational learning allows for word learning to occur across multiple exposures, with a degree of situated cognition across learning instances. For example, Smith and Yu (2008) found that infants rapidly learn multiple word-referent pairs across multiple scenes rather than within a single trial. But there is so much more in the face-to-face caregiver-child interactions than statistical regularities.

Evidence has also been provided that external multimodal cues (such as pointing gestures, eye gaze, iconic gestures, and onomatopoeias) produced by caregivers can support language development. In spoken language, these multimodal cues, along with linguistic input, can draw attention to words, draw attention to the referents, and link a word with its referent (Cartmill et al., 2013; Laing, 2014; McMurray et al., 2022; Nomikou & Rohlfing, 2011; Özçalışkan & Goldin-Meadow, 2005; Perniss & Vigliocco, 2014; Rowe et al., 2008). In combination with discourse context (what has been talked about before) and the physical context (e.g., objects, settings being talked about) of the communication, these multimodal cues can support language acquisition by contributing to the development of embodied representations. Further, context (such as visual scenes) has been shown to improve object recognition and categorization specifically for categories at a superordinate level (Bar, 2004; Borghi et al., 2005), suggesting that for language to become embodied, it must be first situated. The physical presence of the objects, or events, talked about is not strictly necessary to achieve grounding, however. When talking about something displaced, the caregiver imagistically brings attention to the referents by using iconic vocalisations and gestures that imagistically resemble sensorimotor properties of referents (Motamedi et al., 2022). The use of iconic behaviours can provide a powerful tool to ensure that the grounding is possible even when the actual referents are not present in the physical setting (Motamedi et al., 2022; Murgiano et al., 2021).

Moreover, children themselves create learning moments. Yu and Smith (2012), using head mounted cameras on the caregiver and on the child showed that when 18-month-old infants interact with objects in play with their parents, they create moments in which a single object is visually dominant. If parents name the object during these moments of bottom-up selectivity, infants can learn the name more successfully than when naming occurs during a less visually selective moment. The manual behaviours of both the caregiver and of the child, during situated interactions also affect learning. Rowe and Goldin-Meadow (2009) showed that the gestures produced by caregivers affect the gestures produced by their children. In turn, the gestures produced by the children predict their later vocabulary: children using more gestures (especially pointing gestures) at 18 months have a richer vocabulary by 42 months. These studies suggest that cues present in the physical and social-communicative environment can favour the grounding of newly acquired words early on to the sensory, motor, and affective properties of referents, thus allowing for internalisation (and therefore embodiment) to develop.

The importance of the physical and social/communicative context to language development is shown in the success of intervention studies that manipulate the context. It has been found that directing the parent to increase utterance input, respond to the child’s lead, and create opportunities for language learning (interactive book reading, direct teaching of vocabulary and narrative skills) can improve language learning outcomes (Beecher & Van Pay, 2020; Burgoyne et al., 2018; Elmquist et al., 2020; Fricke et al., 2017).

The link between “in the moment” behaviours and embodiment becomes clearer as children age. Gómez et al. (2023) investigated the role of embodied action in classroom settings with 216 students (7.5–8.5 years old) enrolled in second and third grade. Students were randomly assigned to two action conditions (performing an action while reading silently on a screen; pantomime while reading aloud) and two control conditions (no-action while reading silently on a screen; no action while reading aloud). The study showed that the comprehension of a complex text (included concepts such as friction, velocity, and force) in addition to learning 10 targeted vocabulary words was facilitated by action. Importantly, the study showed that action independent of the type, either technology-based action (students reading and performing targeted action on the screen) or action using the whole body (students reading aloud and performing pantomime) can be applied as a successful action-based teaching approach in the classroom. Similarly, Glenberg et al. (2007) found that reading comprehension skills could be enhanced in grade 1 classrooms using a manipulation strategy that included props related to the story (farm toys). Further, de Koning, Bos, et al. (2017) instructed pupils within the classroom to simulate both the visual and motor aspects of a situation in a sentence and apply it to improve reading comprehension. In comparison to a control group, the training group learned strategies to draw upon their physical and sensory experiences and memories to create mental representations of the text they were reading. The children who received the training showed improved reading comprehension and scored higher in motivation to read.

Similarly, Gómez and Glenberg (2021) investigated the role of two types of action while reading an informational text on tablet devices using the EMBRACE application. In this study, 7.5–8.5 years old children, randomly organised in small groups, performed actions that matched selected sentences in the text. The first type of action involved children moving images on a tablet screen. The second type involved children pantomiming the sentences with their own bodies. Finally, there was a control (read-only) no-action condition. They found that both action conditions (either action performed on the screen or pantomimed) significantly improved reading comprehension and vocabulary learning when compared to the no-action condition. These results are consistent with embodied theories. However, it should be noted that other research demonstrates that the recruitment of sensorimotor states does not always boost children’s verb learning, as compared to other non-embodied techniques (e.g., Schwarz et al., 2017) and that the read-only control in the Gómez and Glenberg’s study above does not control for differences in motivation and attention between the conditions in which children performed actions and the one in which they simply read.

Thus, as the research summarised in this section shows, the specifics of the context in which language is learnt matters. We argue that for the perceptual (Guellaï et al., 2014), sensorimotor (Smith, 2005; Wellsby & Pexman, 2014b), and emotional (Lund et al., 2019; Ponari et al., 2018) properties of a concept to support language acquisition, these must be internalised from direct experience as the survey above illustrates.

## Implications for developmental disorders

Developmental difficulties seemingly isolated to one specific ability can impact all areas of development. For example, developmental disorders affecting motor ability (e.g., developmental coordination disorder; DCD) or language skills (e.g., developmental language disorder; DLD) are an important testing ground for theories of embodied and situated cognition. Based on the theoretical conceptualization of embodied and situated concept development, we would expect that difficulty with action-based experiences would impact language development, thus providing insight into this relationship. Indeed, one third of children with DLD are diagnosed with DCD (Albaret & de Castelnau, 2009; Flapper & Schoemaker, 2013) and are reported to have overlapping difficulties (Albaret & de Castelnau, 2009), suggesting that motor ability and language skills are related. Leonard (2016) interpreted these findings to conclude that the motor difficulties of children with DCD will limit their perceptual experience and action with their surroundings, impacting the way their environment is perceived and processed. If learning is facilitated by interaction with the environment, as we argue, children with DCD and other motor difficulties are at a distinct disadvantage.

Due to these overlapping weaknesses in motor skills and language abilities, the research findings have implications for intervention. By scaffolding the child’s learning environment with embodied cues, researchers, practitioners, teachers, and caregivers can provide children with developmental disabilities with additional cues to facilitate concept acquisition. Gómez et al. (2023), as stated previously, found that action facilitated learning for neurotypical children. To examine whether an embodied intervention could benefit individuals with developmental weaknesses, Labrecque et al. (2021) found that some adolescents with DLD seem to have the potential to use movement in the learning process, but not all. They used a grip force modulation technique (Frak et al., 2010) and asked adolescents with DLD and a control group to participate. Adolescents were asked to listen to words that were either related or unrelated to an action while they were holding a cylinder with an integrated force sensor. Previous research has indicated that there is a relationship between language and grip force which helps identify sensorimotor involvement (da Silva et al., 2018; Frak et al., 2021; Frak et al., 2010; Juárez et al., 2019, see also reference above to Macedonia et al., 2020). The grip force modulation is believed to be a hand muscle activity due to hand motor network activated from linguistic processing. The control group presented with a grip force modulation in their dominant hand after they listen to hand-action verbs, but in the DLD group, only half showed grip force modulation. It should be noted that Labrecque et al. (2021) studied adolescents rather than children. More research is needed to determine whether an embodied and situated context can help facilitate learning in younger, pre-adolescent children with developmental disorders.

## Conclusion

We have argued that by clearly defining embodied (internal mechanisms responsible for grounding language in bodily experience) and situated (those mechanisms that dynamically allow for the integration of language with external factors) cognition, the roles of perception, action, and valence in language acquisition can be more clearly understood. Language acquisition, after all, takes place in a complex, interactive environment with multiple speakers, clues from the environment, and the unique language experience of each individual child. Words are presented to the child in a complex environment with many other discourse, physical, and social cues that impact learning. Learning words and concepts takes advantage of the range of external contextual factors that together make language situated, and it is this situatedness that gives rise to embodied effects. More specifically, the paper argued that: 1) language becomes embodied because of the characteristics of the contexts typical of language development. By investigating the contextual variables in which learning occurs we can more closely understand in what way internal representations are embodied as these embodied aspects must reflect the contextual characteristics; 2) More generally, to better understand language processing across childhood and adulthood, the context must be taken into account as differences in context may give rise to differences in embodiment effects (Murgiano et al., 2021; Yee & Thompson-Schill, 2016; Zwaan, 2014). This would allow an increased applicability to the home environment, classroom, and for children identified with language learning disorders.

In some ways, the discussion of the relationship between situated and embodied language relates to the broader debate in cognitive science between episodic and semantic memory (see, De Brigard et al., 2022). Episodic and semantic memories complement each other, but they also interact. Episodic memories provide rich information for semantic generalisations and, hence, are relevant for the formation and modification of semantic memories (Maguire & Mullally, 2013; Moscovitch et al., 2006; Sheldon & Moscovitch, 2012) and semantic memories may affect the encoding of episodic experience (Renoult & Rugg, 2020). In adults, the access to episodic and semantic memories overlaps. For example, a certain word occurring in close temporal relation with a specific object may be encoded and retained as a unique event in episodic memory, while, at the same time, it may represent the labelling of a more general category stored in semantic memory. Only very few studies have investigated the development of the relationship between episodic and semantic memory in young children (Friedrich et al., 2020). Situated language processing can contribute to both episodic as well as semantic memory, as illustrated in the example (the contribution of experiencing a label-object association to both developing an episodic memory for the event as well as providing further generalizability for its semantic memory). Assuming embodied semantic representations, if different situations in which we encounter the object involve specific patterns of interactions, generalisation from the specific situation (episode) will give rise to embodied semantic memories. More generally, the integration of episodic and semantic memory with embodied experiences highlights the importance of context in language acquisition. By experiencing objects in specific situations and contexts, language learners are able to form more detailed and richer representations that allow for an easier and more accurate retrieval of meaning. This emphasises the importance of providing learners with opportunities to engage in embodied experiences that are situated in relevant physical and social contexts. By doing so, we shall help language learners develop a more integrated and contextualised understanding of the world.

While researchers can focus more on either the embodied or the situated aspect of cognition, the two are by nature not independent of each other. Cognition becomes embodied because of its situatedness in specific contexts (i.e., home, school) where learning occurs.
